# Self-reported gender differentials in the knowledge of tuberculosis transmission and curative possibility using national representative data in Ghana

**DOI:** 10.1371/journal.pone.0254499

**Published:** 2021-07-12

**Authors:** Michael Boah, Mary Rachael Kpordoxah, Martin Nyaaba Adokiya

**Affiliations:** 1 Department of Epidemiology, Biostatistics and Disease Control, School of Public Health, University for Development Studies, Tamale, Ghana; 2 Department of Global and International Health, School of Public Health, University for Development Studies, Tamale, Ghana; Federal University of Rio de Janeiro, BRAZIL

## Abstract

**Background:**

Health-seeking behaviour, stigma, and discrimination towards people affected by tuberculosis (TB) are influenced by awareness of the disease. Gender differentials in the diagnosis and treatment of TB have been reported in other settings of the world. However, little is known about the gender differences in the knowledge of TB transmission and curative possibility in Ghana.

**Methods:**

The analysed data were a weighted sample of 9,396 women aged 15–49 years and 4,388 men aged 15–59 years, obtained from the 2014 Ghana Demographic and Health Survey. The dependent variable, correct knowledge regarding TB transmission and cure was derived from questions on the transmission of the disease and the possibility of a cure. A design-based multivariate logistic regression model in Stata 13.0/SE was used to identify the correlates of reporting correct knowledge.

**Results:**

Overall, the mean knowledge score was 6.1±0.9 (maximum = 7). Of the 13,784 respondents, 45.7% (95% CI: 44.0–47.3) reported correct knowledge regarding TB transmission and cure. Men had significantly higher knowledge than women (50.9% versus 43.2%). Misconceptions, including TB transmitted through sharing utensils (13.3%), food (6.9%), touching a person with TB (4.5%), sexual contact (4.1%), and mosquito bites (0.4%) were noted. About 30% (33% women and 25% men) of the total sample would keep the information secret when a household member is affected with TB. In the adjusted analysis, age, gender, education, region, place of residence, wealth quintile, frequency of reading newspaper/magazine, listening to the radio, and watching television were significantly associated with reporting correct knowledge.

**Conclusions:**

There was low knowledge regarding TB transmission and cure. Misconceptions regarding the transmission of TB prevailed among the participants. Gender differential in knowledge was observed. Comparatively, females were less likely to be aware of TB and report correct knowledge regarding TB transmission but were more likely to conceal information when a household member was affected by the disease.

## Introduction

Tuberculosis (TB) is a communicable disease and a major cause of morbidity and mortality. Globally, recent data show significant declines in the burden of TB compared to a decade or two ago. Nevertheless, the disease continues to be a serious public health threat. TB is the leading cause of death from a single infectious agent. In 2019, an estimated 10 million people fell ill with TB, and more than 1 million TB deaths were recorded among people infected with human immunodeficiency virus (HIV) and HIV-negative persons. The burden of TB varied widely by geographical region, most people who developed the disease were in the World Health Organization (WHO) regions of South-East Asia (44%), Africa (25%) and the Western Pacific (18%), with smaller percentages in the Eastern Mediterranean (8.2%), the Americas (2.9%) and Europe (2.5%) [1]. With timely diagnosis and appropriate treatment, TB can be cured and mortality averted. The international community has committed to “Ending TB” as a global epidemic by 2030, backed up by milestones and ambitious targets. The DOTS (Directly Observed Treatment Short-course) system has been used by most low-income and middle-income countries (LMICs) for the control of TB. The implementation of this strategy has yielded desirable results in some countries such as China and Peru, while in some countries, particularly those of sub-Saharan Africa (SSA), the strategy has not yielded the desired outcome and impact, in part, due to the high prevalence of HIV [2].

The National TB Control programme (NTP) of Ghana was instituted in 1994 to oversee TB control activities in the country after a period of neglect. The DOTS strategy for the control of TB was also implemented in the same year [3, 4]. With funding from the Danish International Development Agency (DANIDA) and Global Fund, TB control activities in Ghana were strengthened after a period of neglect, including the expansion of DOTS activities to cover 100% of the population in 2005. As a result, significant improvements in TB treatment success rates have been reported on the one hand, while on the other hand. However, after nearly three decades following the implementation and expansion of DOTS in the country, TB treatment coverage has consistently been far below the international target of 70%. According to the Global TB reports, the TB treatment coverage and treatment success rate of Ghana averaged 28.7% (range: 13–34%) and 70.4%(range: 50–85%) respectively, between 1995 and 2019 [1]. More broadly, the under-notification of TB cases is a challenge in many LMICs and many millions of patients have been missing out on diagnosis and treatment. Moreover, the DOTS system in improving TB case detection is limited by its design. This is because DOTS mainly employs a passive system of case identification, which requires patients with symptoms of TB to self-report to health institutions for diagnosis [5].

At the global level and in most countries, including Ghana, TB case notification rates are higher among men than women [1]. The reasons behind this observation are not clearly understood. Plausible explanations from scholars suggest that the differences are likely from a complex mixture of factors such as different risk factors and different health-seeking behaviour for diseases between men and women [6–9]. Some findings have also argued that men and women have equal access to TB diagnosis and treatment, therefore, the observed gender differences in TB notifications were unlikely to result from gender inequalities in access to health care services [10]. However, it is widely recognized that knowledge about diseases influences health-seeking behaviour. Reports from Asia, specifically, India, have demonstrated the impact of TB disease awareness on voluntary reporting at health facilities for TB care [11]. Generally, significant patient delays in TB diagnosis and non-seeking of care have been associated with a lack of knowledge and misconceptions about TB disease [12, 13].

Little is known about gender differences in TB knowledge among men and women. Results of studies conducted among TB patients to assess knowledge on the disease, including signs, cause, and mode of transmission, as well as the possibility of cure have been inconclusive [14, 15]. Furthermore, large population-based studies have also shown that knowledge about TB disease in the general population of countries highly infected with the disease is low, ranging from 16% in China to 44% in Ethiopia [8, 16, 17]. Poor knowledge about TB and misconceptions about its transmission are important drivers for delayed diagnosis and treatment as well as stigma towards TB patients. Therefore, there is a need to influence people’s knowledge, behaviours, and attitudes to enable them to make healthy lifestyle choices. More specifically, an understanding of people’s knowledge on the transmission of TB could help in the design of programmes to address misconceptions and eliminate, if not, reduce the discrimination towards TB patients in Ghana [18].

The purpose of the present study was to examine the gender differences in knowledge regarding TB disease transmission and cure and identify the correlates of self-reported correct knowledge regarding TB transmission and cure in Ghana using nationally representative data.

## Methods

### Data source

This study analyzed data obtained from the 2014 Ghana Demographic and Health Survey (GDHS), the sixth in a series of population and health surveys conducted in Ghana as part of the global Demographic and Health Surveys (DHS) Program. The survey was implemented by the Ghana Statistical Service (GSS), the Ghana Health Service (GHS), and the National Public Health Reference Laboratory (NPHRL) of the GHS. An updated frame from the 2010 Population and Housing Census conducted in Ghana was used as the survey sampling frame for the 2014 GDHS. Nomads and persons in hotels, barracks, and prisons were excluded from this sampling frame. A two-stage sample strategy was used in the survey to select 427 clusters consisting of enumeration areas (216 in urban areas and 211 in rural areas) and households. About 30 households were selected from each cluster to constitute the total sample size of 12,831 households. Because of the approximately equal sample sizes in each region, the sample is not self-weighting at the national level, and weighting factors have been added to the data file so that the results will be proportional at the national level. All women aged 15–49 who were either permanent residents of the selected households or visitors who stayed in the household the night before the survey were eligible to be interviewed. On the other hand, in half of the households, all men aged 15–59 who were either permanent residents of the selected households or visitors who stayed in the households the night before the survey were eligible to be interviewed [19].

Overall, 12,831 households were selected for the sample, of which 12,010 were occupied. Of the occupied households, 11,835 were successfully interviewed (99% response rate). Of the 9,656 eligible women identified for individual interviews from the interviewed households, interviews were completed with 9,396 women (97% response rate). In the subsample of households selected for the male survey, 4,609 eligible men were identified and 4,388 were successfully interviewed (95% response rate). For the present study, data on 13,784 Ghanaians (9,396 women and 4,388 men) were analyzed by merging the women’s and men’s files.

### Outcome and exposure variables

Information on participant’s knowledge and attitude towards TB was collected in the 2014 GDHS. Participants were asked whether they had ever heard of TB, the mode of transmission, whether it can be cured, and whether they would want to keep the information secret if a member of their family contracted the disease. The respondents’ knowledge of tuberculosis transmission was elicited by asking, "How does tuberculosis spread from one person to another?" Participants were permitted to mention more than one response. For the present study, the response categories were: 1) through the air when coughing or sneezing; 2) through sharing utensils; 3) through touching a person with TB; 4) through food; 5) through sexual contact; 6) through mosquito bites; 7) others, and 8) don’t know. Because multiple responses were allowed, a *“Yes”* for any option mentioned and a “*No”* for any option not mentioned. A dependent variable “Knowledge regarding TB transmission and cure” with a binary outcome (“1”: has correct knowledge regarding TB transmission and cure and “0”: has misconceptions regarding TB transmission and cure) was created from the responses of participants to the question on TB transmission and the possibility of cure. For our analyses, a score of “1” point was assigned to each correct response. To be able to compute the percentage of respondents who had knowledge about TB transmission without any associated misconceptions, we also assigned “1” points to the other incorrect responses (misconceptions) about the spread of TB that were not mentioned. Thus, the respondent scored “1” point for mentioning the first option *“through the air when coughing or sneezing”* and “1” point for answering no to each of the other options (i.e. misconceptions) on mode of transmission. A respondent who mentioned an incorrect response was given a score of “0” points. The scores on TB transmission ranged from a minimum of 0 to a maximum of 6 points. There were no responses under the “Others” and “Don’t know” categories. Lastly, “1” point was also given to respondents who answered in the affirmative to the question on whether TB can be cured. In this study, a respondent was classified as having correct knowledge about TB transmission and cure (i.e. without any misconceptions) if he/she obtained all 6 points on TB transmission and 1 point on the possibility of cure, giving a total of 7 points. Respondents with less than 7 points had other misconceptions about the spread of tuberculosis, regardless of whether they knew the disease was curable or spread through the air. Such respondents were classified in the category “had misconceptions regarding TB transmission and cure”. We used information on keeping TB status hidden if a household member contracted the disease as a proxy to assess the stigma associated with the disease. Participants were asked the question “If a member of your family got tuberculosis, would you want it to remain a secret or not?” The options available were; 1) Yes, remain a secret; 2) No, and 3) Don’t know/not sure/depends. Participants who responded “Yes, remain a secret” were assumed to be aware of TB-related stigma and that influenced their choice of response.

Some variables were selected from the dataset as predictor variables and included age, education, employment, place of residence (rural or urban), region of residence, wealth quintile, cigarette smoking status, and variables measuring exposure to media. In the 2014 GDHS, exposure to media was assessed by asking respondents whether they read a newspaper or magazine, listened to the radio, or watched television at least once a week. Responses were classified as “Not at all” for respondents who reported that they did not read newspaper/magazine, listen to the radio, or watch television at least once a week. On the other hand, a response was grouped under “At least once a week” if the respondents self-reported that they read newspaper/magazine, listened to the radio, or watched television at least once a week. The wealth quintile was constructed from household asset data using principal component analysis [20]. These assets include a television, bicycle, or car, as well as home characteristics such as a source of drinking water, sanitation facilities, and flooring material type. The inclusion of predictor variables in this study was guided by previously published data on factors associated with TB knowledge [13, 21, 22].

## Data analysis

The data were analyzed using Stata/SE 13.0 (StataCorp, College Station, Texas 77845, USA). The student t-test was used to compare the means of continuous variables between men and women. The differences in categorical variables examined used Pearson’s design-based Chi-square test. A probability value (p-value) of <0.05 was considered statistically significant. A design base multivariable binary logistic regression analysis with robust standard errors was used to assess the relationship between the dependent variable, knowledge regarding TB transmission and cure and age, gender, educational level, employment, region, place of residence, wealth quintile, cigarette smoking, reading newspaper/magazine, listening to the radio, and watching television as predictor variables. The adjusted odds ratio (AOR), their corresponding 95% confidence intervals (CIs), and p-values were reported. The fit of the model was tested using the Archer and Lemeshow goodness-of-fit test for a logistic regression model fitted with survey data [23]. We did not find enough statistical evidence of a lack of fit of our model (F (9, 399) = 0.743; p = 0.669). Weighting was used in our analyses to account for the unequal probability sampling techniques used by the demographic and health program in the surveys to expand the number of cases available (and hence reduce sample variability) for certain areas or subgroups for which statistics are needed.

## Ethical considerations

This study was a secondary analysis of survey data and did not require approval by the Institutional Review Board (IRB). However, the protocol for the 2014 GDHS was reviewed and approved by the Ghana Health Service Ethical Review Committee and the Institutional Review Board of ICF International.

## Results

### Background information of respondents in this study

From the total of 13,784 participants, 68.2% were women aged 15–49 years, whereas 31.8% were men aged 15–59 years. The background characteristics of the respondents have been presented in Table 1. There were significant disparities between men and women by age, educational level, employment, place of residence, cigarette smoking, and exposure to the three forms of media. The results showed that 75.5% of men had received at least secondary level education compared with 63.1% of women (p<0.001). In addition, 13.7% of men were unemployed compared with 23.5% of women. Regarding exposure to media, 35% of men reported reading newspapers/magazines at least once a week compared with about 19% of women. On the other hand, about 16% and 23% of women reported not listening to radio and television, respectively, compared with about 6% and 17% of men who reported not listening to radio and television, respectively. The differences between men and women by wealth were not statistically significant (Table 1).

**Table 1:** Background information of respondents in this study (N = 13,784).

Variable	Total (N = 13,784)	Women (n = 9,396)	Men (n = 4,388)	P-value
	Weighted %	Weighted %	Weighted %	
**Age (Years)**				
Mean ±SD	30.7±10.6	29.9±9.6	32.4±12.3	<0.001
15–24	33.9	34.4	32.9	
25–34	29.9	31.7	26.0	
35+	36.2	33.9	41.1	
**Education**				<0.001
No formal education	16.4	19.1	10.7	
Primary	16.4	17.8	13.5	
Secondary	59.1	56.8	64.0	
Higher	8.1	6.3	11.8	
**Employment**				<0.001
Unemployed	20.4	23.5	13.7	
Employed	79.6	76.5	86.3	
**Region**				0.667
Greater Accra	20.5	20.2	21.0	
Western	11.2	11.1	11.4	
Central	9.9	10.0	9.6	
Volta	7.7	7.7	7.7	
Eastern	9.5	9.3	9.8	
Ashanti	18.8	19.1	18.0	
Brong Ahafo	8.2	8.2	8.3	
Northern	8.3	8.4	8.1	
Upper East	3.8	3.8	3.8	
Upper West	2.3	2.3	2.3	
**Place of residence**				0.043
Urban	53.2	53.8	52.1	
Rural	46.8	46.2	47.9	
**Wealth quintile**				0.140
Poorest	16.4	16.1	17.1	
Poorer	17.5	17.4	17.8	
Middle	20.1	20.6	19.0	
Richer	22.3	22.5	21.9	
Richest	23.6	23.4	24.2	
**Smokes cigarettes**				<0.001
No	98.4	99.9	95.2	
Yes	1.6	0.1	4.8	
**Reads newspaper/magazine**				<0.001
Not at all	75.9	81.1	64.7	
At least once a week	24.1	18.9	35.3	
**Listens to radio**				<0.001
Not at all	12.4	15.6	5.5	
At least once a week	87.6	84.4	94.5	
**Watches television**				<0.001
Not at all	21.3	23.4	16.8	
At least once a week	78.7	76.6	83.2	

### Gender differences in TB awareness, knowledge of its transmission and cure, and stigma

The gender differences in TB awareness, knowledge regarding TB transmission, cure, and stigma have been presented in Table 2. The analysis demonstrated that 85% of the total sample had heard about TB as an illness (82.9% women and 89.4% men). Regarding the mode of transmission of the disease, 67.2% reported that TB is spread through the air when coughing or sneezing. There were also misconceptions reported by the participants on TB disease transmission, including that it spreads through sharing utensils (13.3%), touching a person with TB (4.5%), food (6.9%), sexual contact (4.1%), and mosquito bites (0.4%). About 86% of the total participants reported that TB is curable, while approximately 7% said they “don’t know”. We noted statistically significant gender differences in the responses to TB transmission and cure between men and women. For instance, the percentage of men who reported that TB is spread through the air when coughing or sneezing was higher than that of women (72% versus 65%; p<0.001). Furthermore, more men than women reported that TB can be cured (90% versus 85%; p<0.001) (Table 2).

**Table 2:** Gender differences in tuberculosis awareness, knowledge regarding disease transmission and cure, and stigma (N = 13,784).

Indicator	Total sample (N = 13,784) Weighted %	Women (n = 9,394) Weighted %	Men (n = 4,388) Weighted %	P-value
**Heard about TB**				<0.001
No	15.0	17.1	10.6	
Yes	85.0	82.9	89.4	
**TB transmission; TB spread by:**				
**Air when coughing or sneezing**				<0.001
No	32.8	35.0	28.1	
Yes	67.2	65.0	71.9	
**Sharing utensil**				0.125
No	86.7	86.2	87.9	
Yes	13.3	13.8	12.1	
**Touching a person with TB**				0.082
No	95.5	95.8	94.9	
Yes	4.5	4.2	5.1	
**Food**				0.012
No	93.1	93.6	92.1	
Yes	6.9	6.4	7.9	
**Sexual contact**				0.005
No	95.9	95.5	96.9	
Yes	4.1	4.5	3.1	
**Mosquito bites**				0.007
No	99.6	99.5	99.8	
Yes	0.4	0.5	0.2	
**Cure**				
**TB can be cured**				<0.001
No	7.2	8.2	5.1	
Yes	86.2	84.5	89.7	
Don’t know	6.6	7.3	5.2	
**TB stigma**				
**Keep secret when a family member gets TB**				<0.001
No	68.3	65.1	74.5	
Yes, remain a secret	29.9	32.6	24.7	
Don’t know	1.8	2.3	0.8	
**Correct knowledge of TB transmission and cure (i.e. without any misconceptions)**	45.7	43.2	50.9	<0.001
TB knowledge score; Mean± SD	6.1 ±0.9	6.1 ±1.0	6.2± 0.9	<0.001

Based on the operational definition of correct knowledge regarding TB transmission and cure used in this study, the results showed that overall, knowledge regarding TB transmission and cure was correctly reported by 6,295 (45.7%; 95% CI: 44.0–47.3) participants. Similarly, knowledge regarding TB transmission and cure was disproportionately distributed between men and women in the present study. Our data showed that 50.9% (95% CI: 48.2–53.7) of men reported correct knowledge regarding TB transmission and cure, whereas 43.2% (95% CI: 41.4–45.0) of women reported correct knowledge regarding TB transmission and cure. We also examined the attitude of respondents towards TB disease as a proxy for measuring stigma associated with the disease. The results showed that in the total sample, about 30% of participants would keep the information secret if a family member contracted the disease. There was a statistically significant difference by gender in the attitude towards keeping TB status in the family secret; it was observed that women were more likely than men to report that they would keep the information secret (33% versus 25%; p<0.001) (Table 2).

### Determinants of correct knowledge regarding TB transmission and cure in Ghana

A survey-based multivariate binary logistic regression model was used to identify the determinants of reporting correct knowledge regarding TB transmission and cure among the study participants. The results are presented in Table 3. We found that age, gender, education, region, place of residence, wealth quintiles, frequency of reading newspaper/magazine, listing to the radio, and watching television were statistically significantly correlated with knowledge regarding TB transmission and cure.

**Table 3:** Determinants of correct knowledge regarding tuberculosis transmission and cure in Ghana.

Age group (Years)	AOR (95% Confidence interval)	P-value
15–24	1	
25–34	1.31(1.15–1.49)	<0.001
35+	1.14(1.01–1.30)	0.038
**Gender**		
Women	1	
Men	1.20(1.05–1.38)	0.010
**Educational level**		
No formal education	1	
Primary	0.96(0.83–1.12)	0.607
Secondary	1.72(1.48–2.00)	<0.001
Higher	2.32(1.76–3.08)	<0.001
**Employment**		
Unemployed	1	
Employed	1.02(0.90–1.16)	0.737
**Region**		
Greater Accra	1	
Western	0.83(0.66–1.05)	0.120
Central	1.16(0.94–1.43)	0.169
Volta	0.98(0.74–1.30)	0.885
Eastern	0.90(0.73–1.12)	0.361
Ashanti	1.15(0.95–1.41)	0.156
Brong Ahafo	0.76(0.60–0.96)	0.021
Northern	0.92(0.72–1.19)	0.521
Upper East	1.03(0.70–1.51)	0.872
Upper West	0.77(0.52–1.16)	0.208
**Place of residence**		
Urban	1	
Rural	0.83(0.72–0.96)	0.010
**Wealth quintile**		
Poorest	1	
Poorer	1.02(0.86–1.22)	0.803
Middle	1.12(0.92–1.36)	0.250
Richer	1.36(1.10–1.67)	0.004
Richest	1.62(1.27–2.06)	0.000
**Smokes cigarettes**		
No	1	
Yes	0.73(0.50–1.06)	0.094
**Reads newspaper/magazine**		
Not at all	1	
At least once a week	1.34(1.19–1.52)	<0.001
**Listens to radio**		
Not at all	1	
At least once a week	1.40(1.21–1.61)	<0.001
**Watches television**		
Not at all	1	
At least once a week	1.25(1.09–1.42)	0.001

AOR: adjusted odds ratio (adjusted for the other variables included in the analysis)

From the results (Table 3), participants in the age group of 25–34 (AOR = 1.31; 95% CI: 1.15–1.49) and those who are ≥35 years (AOR = 1.14; 95% CI: 1.01–1.30) had increased odds of reporting correct knowledge compared to participants in the age group of 15–24 years. The likelihood of reporting correct knowledge was 20% higher among men than among women (AOR = 1.20; 95% CI: 1.05–1.38). The likelihood of reporting correct knowledge was also statistically significantly higher among participants with secondary (AOR = 1.72; 95% CI: 1.48–2.00) and tertiary education (AOR = 2.32; 95% CI: 1.76–3.08) compared to those with no formal education. Participants in the richer (AOR = 1.36; 95% CI: 1.10–1.67) and richest (AOR = 1.62; 95% CI: 1.27–2.06) wealth quintiles had significantly higher odds of reporting correct knowledge compared with women in the poorest wealth quintile. Statistically significant odds were noted among participants who reported reading newspaper/magazine at least once a week compared to those who did not read at all (AOR = 1.34; 95% CI: 1.19–1.52). The results were similar for listening to the radio and watching television.

Conversely, lower odds of reporting correct knowledge were observed among people residing in rural areas compared to urban dwellers (AOR = 0.83; 95% CI: 0.72–0.96). Similarly, compared to respondents from the Greater Accra region, respondents from the Brong Ahafo region were statistically significantly less likely to report correct knowledge regarding TB transmission and cure (AOR = 0.76; 95% CI: 0.60–0.96) (Table 3).

## Discussion

The analyses revealed that the majority (85%; 83% women, 89% men) of the participants had heard about tuberculosis (TB). This implied a high awareness about TB in the Ghanaian population, which was within the range reported (83% and 94%) by studies conducted in Ethiopia [13, 24]. More than half of the participants also knew that the disease was spread through the air when coughing or sneezing. However, some respondents reported some misconceptions, such as sharing utensils, touching a TB patient, food, sexual contact, and mosquitoes. These findings are similar to other studies, which reported that although the majority of people in the population have heard about TB, there were misconceptions (e.g. sharing drink, sharing food, touching an infected person, etc.) regarding the mode of transmission [16, 24, 25]. In this study however, smaller percentages were observed for misconceptions compared with reports from other places. For instance, Pakistan where about 44% and 32% of participants identified sharing utensils and food respectively, as sources of TB transmission [25]. Notably, in the present study, more than 8 in 10 participants knew that TB is curable, albeit significant differences in knowledge between men and women.

When the analysis applied the operational definition of correct knowledge regarding TB transmission and cure, which is knowledge of the mode of transmission without any form of misconceptions, we found that overall, the knowledge was low; less than half of the sample (45.7%) had correct knowledge on the disease transmission and possibility of cure. Furthermore, men had significantly higher odds than women to report correct knowledge, which may have been influenced by their higher educational level and greater exposure to media compared to women. This explanation is supported by the Ghana Statistical Service report [26]. Our findings on low knowledge regarding the transmission of TB in the population and among females compared with males are consistent with the findings of Sreeramareddy and colleagues who reported that about 30% of Indians had correct knowledge about TB transmission without misconceptions and males were more likely to have correct knowledge than females [16]. Conversely, an adjusted analysis from a study in Nigeria, which measured TB knowledge using cause, routes of transmission, and methods of prevention found weak statistical evidence that females were more likely than males to have good knowledge about TB [27]. Partly, the variation in findings may have resulted from the differences in measuring knowledge in this study and the Nigeria study.

The results showed that a high percentage of the participants would keep TB information secret within the household when a member contracts the disease. TB is a disease associated with poverty. Stigma and discrimination against people with TB are common in many settings, particularly in poor areas. In Ghana, for the fear of infection, physical distancing and participatory restrictions are placed on people affected by TB. Community members hold the view that affected persons should not be involved in some social activities, such as selling in the market [18]. In this study, we found that females were significantly more likely than males to report that they would keep the information concealed when a household member contracted TB. The likely explanation may be related to the vulnerability of females than males to the social consequences of TB related stigma [28]. More importantly, evidence from a cross-site study revealed that women were more disadvantaged with regard to self-esteem, social isolation, and perceived stigma compared to men [29]. Regardless, these findings have implications for TB control in Ghana and other LMICs. The fear of being stigmatized, discriminated against, or socially isolated may force TB patients and/or their families to exhibit inappropriate health-seeking behaviours. TB patients or people who suspect they have the disease may intentionally hide their status or attribute their symptoms to non-stigmatizing disease conditions, which may intend lead to delayed diagnosis and treatment and spread of the disease in the population. It may also lead to persistent underreporting of TB cases among females. Therefore, there is the need for intensified public education on TB, particularly the mode of transmission, to reduce negative attitudes towards people affected by the disease at the community-level.

The analyses also identified some correlates of correct knowledge regarding TB disease transmission and cure. Consistent with previous reports, we found that age was significantly associated with reporting correct knowledge. The results demonstrated that, compared to persons aged 15–24 years, persons older than 24 years were more likely to report correct knowledge. In addition, the results highlighted that education and wealth were important correlates of correct knowledge. Comparatively, participants who had at least secondary level education and participants in the richest wealth quintile were more likely to report correct knowledge. These findings support earlier reports from Tanzania [30], India [16], Ethiopia [24], and Nigeria [27]. By administrative region, the results revealed that participants from the Brong Ahafo region of Ghana were less likely than those in the Greater Accra region to report correct knowledge. Although the reasons for this variation are not clearly understood, it’s unlikely that the differences in literacy rate and exposure to mass media between the two regions do not provide a plausible explanation [26].

From the results, rural participants were less likely than urban residents to report correct knowledge. Existing data show that knowledge regarding all aspects of TB was particularly deficient in rural areas than in urban areas [31]. Rural dwellers are more likely to be less educated and less exposed to health information compared to urban dwellers. Therefore, knowledge about TB transmission in rural areas may be characterized by more misconceptions. A national study reported that respondents from rural areas were more likely to report that TB spreads through touching infected persons and through mosquito bites compared to those from urban areas [30]. More generally, rural populations have poor access to health information and care, delay seeking care for TB, and are more likely to have TB patients in the family, and should be targeted with interventions to increase awareness on TB [12, 31, 32].

Finally, the results showed that exposure to media such as newspapers, television, and radio was positively related to reporting correct knowledge about TB disease transmission and cure. These findings align with an earlier study, which reported a significant association between the frequency of listening to the radio and correct knowledge regarding TB transmission [16]. The frequency of reading newspaper/magazines and watching television were not significantly associated with the correct knowledge on TB transmission in the same study. The media are a source of TB information as established elsewhere [33, 34]. In Ghana, household possession of television has increased in recent years. For instance, the percentage of households that owned television increased from 43% in 2008 to 62% in 2014 [19]. From the most recent Ghana Multiple Indicator Cluster Survey (20017/18), the percentage of the population exposed to any form of media has also increased appreciably (e.g., 69% of women in 2014 compared to 77.1% of women in 2018) [26]. These media channels could be used to disseminate information on TB that would increase awareness at the population level and contribute to reducing discrimination and stigma associated with TB.

## Study limitations

Although this study has presented findings that are beneficial for targeting interventions to increase knowledge of the general population regarding the transmission of TB and reduce stigma and discrimination among TB patients, there are some limitations. Firstly, this study relied on secondary data from the 2014 GDHS. The questionnaire used only gathered information relating to TB by asking questions on awareness of TB, its mode of transmission, the possibility of cure, and whether respondents would keep the information secret if a household member was affected by TB. Therefore, we had to rely on the available information to create a dependent variable. Information such as signs and symptoms, prevention methods, awareness about DOTS and TB treatment facilities would have added more insights into knowledge about the disease in the general population. Secondly, participants were not asked about their source of information on TB. As a result, by including the variables on media exposure, we are assuming that participants were exposed to information on TB disease from these sources. Indeed, there is evidence to back up our assertion [33, 34]. Lastly, this study is cross-sectional by design, therefore, causal conclusions cannot be drawn.

## Conclusion

This study found a large proportion of the participants had limited knowledge regarding the transmission and cure of TB. Participants’ knowledge regarding the transmission of the disease was limited by misconceptions, including disease spread through sharing utensils, food, touching infected persons, sexual contact, and mosquito bites. Gender difference in the knowledge of TB transmission and cure was observed. Comparatively, females were less likely to be aware of TB and report correct knowledge regarding TB transmission. However, they were more likely to conceal information when a household member was affected by the disease. Rural residents and participants from the Brong Ahafo region of Ghana had lower odds of reporting correct knowledge regarding TB transmission and cure. Exposure to media such as reading newspaper/magazine, listening to the radio, and watching television were positively associated with reporting correct knowledge. Increasing public awareness on TB disease can impact positively on TB knowledge, which may dovetail into positive health-seeking behaviour such as voluntary reporting to health facilities for TB care and reduced stigma towards TB patients, evidenced by findings of the present study and existing reports [11].
